# Identification of RNA silencing suppressor encoded by citrus chlorotic dwarf-associated virus

**DOI:** 10.3389/fmicb.2024.1328289

**Published:** 2024-01-25

**Authors:** Xiao Ye, Dongdong Ding, Yuan Chen, Chuang Liu, Zhongan Li, Binghai Lou, Yan Zhou

**Affiliations:** ^1^Integrative Science Center of Germplasm Creation in Western China (CHONGQING) Science City/Citrus Research Institute, Southwest University, Chongqing, China; ^2^Lemon Industry Development Center, Anyue, Sichuan, China; ^3^Guangxi Citrus Breeding and Cultivation Research Center of Engineering Technology/Guangxi Academy of Specialty Crops, Guilin, Guangxi, China

**Keywords:** citrus chlorotic dwarf-associated virus, RNA silencing, V2 protein, Geminivirus, suppressor

## Abstract

**Introduction:**

Citrus chlorotic dwarf-associated virus (CCDaV) is an economically important citrus virus associated with leaf curling, deformation, and chlorosis found in China. Plants have evolved RNA silencing to defend against viral infections; however, the mechanism by which CCDaV suppresses RNA silencing in citrus remains unknown.

**Methods:**

Six proteins encoded by CCDaV were ectopically expressed in *Nicotiana benthamiana* 16c using the pCHF3 vector to identify RNA-silencing suppression activities.

**Results:**

V2 protein encoded by CCDaV suppressed local RNA silencing and systemic RNA silencing triggered by GFP RNA, but did not impede short-distance movement of the RNA silencing signal in *N. benthamiana* 16c. GFP fluorescence observations showed that the ability of V2 protein to suppress RNA silencing was weaker than tomato bushy stunt virus P19. Deletion analysis showed that the putative nuclear localization signal (NLS, 25–54 aa) was involved in the RNA silencing suppression activity of V2 protein. Furthermore, V2 protein cannot block dsRNA-triggered RNA silencing. The subcellular localization assay suggested that V2 protein was localized to nucleus of *N. benthamiana*.

**Conclusion:**

Overall, the results of this study demonstrate that CCDaV-V2 acts as an activity of silencing suppression. This is the first reported RNA-silencing suppressor encoded by *Citlodavirus* and will be valuable in revealing the molecular mechanism of CCDaV infection.

## 1 Introduction

RNA silencing is a highly conserved regulatory mechanism of gene expression, occurring in almost all eukaryotes. RNA silencing relies on the production of 21–24 nt double-stranded RNA (dsRNA) duplexes, which are one of the important defense systems of plants against viral infection (Pumplin and Voinnet, 2013). Viral small interfering (si) RNA moves from cell to cell systematically limiting the spread of virus in plants (Chen and Rechavi, 2022). To counteract the RNA-silencing defense mechanism, plant viruses encode one or more specific proteins as a RNA-silencing suppressor (RSS) to impede different steps of RNA interference (Lopez-Gomollon and Baulcombe, 2022).

Geminiviruses are the largest group of DNA viruses and cause serious economic losses to a variety of crops globally (Hanley-Bowdoin et al., 2013). Geminiviruses are transmitted by insects, such as whiteflies, leafhoppers, or treehopper species, and are divided into 14 genera (Roumagnac et al., 2022). Among these genera, *Citlodavirus* is a new genus, including closely related viruses isolated from *Citrus* (Loconsole et al., 2012), *Camellia* (Zhang et al., 2018), *Passifora* (Fontenele et al., 2018), and *Broussonetia* (Qiu et al., 2020). C2, Rep, V2/AV2, C4/AC4, C5/AC5, and βC1 encoded by different geminiviruses have been identified as RSS (Bisaro, 2006; Eini et al., 2012; Li et al., 2015). However, no RSS protein has been identified in citlodaviruses.

Citrus fruits are one of the most important fruits worldwide. Recently, a new geminivirus, citrus chlorotic dwarf-associated virus (CCDaV), has been identified in lemon (*Citrus lemons*) in Turkey, which exhibited leaf curling, deformation, and chlorosis symptoms (Garnsey, 1996). Subsequently, it has been detected in Japan, China, and Thailand (Guo et al., 2015; Kasukabe et al., 2017; Yang et al., 2020). CCDaV affects the production of lemon and some pomelo fruits in some citrus-producing provinces in China (Yang et al., 2022). Although the incidence of this disease is currently low, CCDaV poses a potential threat to the Chinese citrus industry. CCDaV is a putative member of the genus *Citlodavirus*, with a highly conserved circular single-stranded DNA genome of approximately 3.64 kb encompassing six predicted open reading frames (ORFs). The sense strand encodes the predicted coat protein (CP) V1, two small hypothetical proteins (V2 and V3), and the putative movement protein (MP) V4 (Roumagnac et al., 2022). Furthermore, RepA (C1) and Rep (C2), which are encoded by the complementary strand, are potential key virulence factors of CCDaV (Qin et al., 2023). A previous study showed that many pathogenicity determinants of viruses also have the function of RSS (Wu et al., 2022), but a CCDaV-encoded silencing suppressor has never been identified.

In the present study, the RSS activity of all proteins encoded by CCDaV was tested using an *Agrobacterium*-mediated transient expression assay in transgenic *Nicotiana benthamiana* 16c. The ability of CCDV-V2 to inhibit gene silencing is weaker than that of tomato bush dwarf viruses (TBSV) P19. The N-terminal 25–54 amino acid (aa) sequence of V2 was involved in suppressing RNA silencing in *N. benthamiana.* V2 could not interfere with the intercellular movement of the RNA silencing signal but could suppress systemic silencing. Furthermore, V2 cannot suppress dsRNA-triggered RNA silencing.

## 2 Materials and methods

### 2.1 Plant materials

Wild-type *N. benthamiana* and transgenic *N. benthamiana* line 16c (kindly provided by Dr. Gentu Wu from Southwest University, China) were grown in an incubator at 25°C under a 16:8-h light/dark cycle.

### 2.2 Plasmid construction

V1, V2, V3, V4, Rep, and RepA of CCDaV isolate (ON063221.1) collected from Chongqing, China, were individually amplified from the infectious clone pBinPLUS-1.6mer using PrimeSTAR Max DNA Polymerase (TaKaRa) and cloned into the pESI-T vector (Yeasen). These plasmids were digested with *Sal*I and *Kpn*I and recombined into the binary vector pCHF3 (kindly provided by Professor Xiuling Yang from the Institute of Plant Protection, Chinese Academy of Agricultural Sciences, China) to generate pCHF3-V1, pCHF3-V2, pCHF3-V3, pCHF3-V4, pCHF3-Rep, and pCHF3-RepA. Constructions were verified by sequencing. Specific primer pairs used in this study are listed in Supplementary Table S1.

### 2.3 Identification of RNA silencing suppressor

### 2.4 Subcellular localization of V2 and its truncates in *Nicotiana benthamiana*

The online software Cell-PLoc 2.0^1^ and cNLS Mapper^2^ were used to predict the subcellular localization and importin α-dependent NLS of V2. Furthermore, full-length V2 was cloned into the *Xba*I site of the pCV-GFP vector to obtain pCV-V2-GFP, as previously described (Yang et al., 2018; Lu et al., 2019). *A. tumefaciens* GV3101 carrying pCV-V2-GFP was co-infiltrated with mCherry-H2B on *N. benthamiana* (Qin et al., 2023). At 2 days post-infiltration (dpi), the co-infiltrated leaves were detected using a FV3000 laser-scanning confocal microscope (Olympus, Japan). To test the role of the putative NLS to the nuclear localization ability of V2, two deletion mutants V2^△25-54aa^ (deletion 25–54 amino acids) and V2^△110-136aa^ (deletion 110–136 amino acids) were constructed.

### 2.5 Conservation and phylogenetic analyses of V2

Multiple amino acid sequence alignments of V2 from CCDaV and 17 RSSs from 7 genera of Geminiviridae family, including tobacco curly shoot virus-V2 (QXJ13566.1), tomato yellow leaf curl virus-V2 (WCA31855.1), tomato golden mosaic virus-AC2 (NP_077737.1), cabbage leaf curl virus-C2 (AWV91967.1) from *Begomovirus*, apple geminivirus1-V2 (YP_009129268.1) from *Maldovirus*, beet curly top virus-V2 (ACB97651.1) from *Curtovirus*, grapevine red blotch virus-V2 (WBA90 091.1) and C2 (WBA90094.1) from *Grablovirus*, mulberry mosaic dwarf-associated virus-V2 (QIK02121.1) from *Mulcrilevirus*, mungbean yellow mosaic India virus-AC2 (NP_803149.1), mungbean yellow mosaic virus-AC4 (UYO78292.1), cotton leaf curl multan virus-C4 (ARR96869.1), tomato yellow leaf curl virus-C5 (QIH13015.1) and βC1 (ACZ71251.1), and squash leaf curl China virus-C5 (UMM62437.1) from *Begomovirus*, and wheat dwarf virus-Rep (QCZ25039.1 from *Mastrevirus*), were performed using SDT v1.2 (Muhire et al., 2014). Phylogenetic analysis of these RSSs was conducted with the MEGA 7 package using the neighbor-joining method (Kumar et al., 2016).

### 2.6 Total RNA extraction and RT-qPCR analysis

Total RNA was extracted from infiltrated leaves of *N. benthamiana* 16c using IsoPlus (TaKaRa, Japan) at 6 dpi based on the manufacturer’s instructions. RT-qPCR was performed using qTOWER3G (Analytik Jena, Germany), as described previously. Relative expression levels were calculated using a 2^-△△Ct^ formula, and *NbActin* gene was used as an internal control (Ananthakrishnan et al., 2010). Primers used for qRT-PCR are listed in Supplementary Table S1. Each experiment was performed in triplicates.

### 2.7 Western blot analysis

Total protein was extracted from approximately 100 mg of the infiltrated leaf samples using a Plant Total Protein Extraction Kit (Solarbio, China), and the extracted protein was used for western blot analysis (Ye et al., 2015; Du et al., 2022). The results were obtained using a chemiluminescence detection kit (Everbright, Beijing, China).

### 2.8 Statistical analyses

GraphPad Prism 9.3.1 was used for statistical analyses. A one-way ANOVA was used for significant analysis. Values were considered significantly different at *p* < 0.05.

## 3 Results

### 3.1 V2 function as RNA silencing suppressors of *GFP*

**Figure 1:**
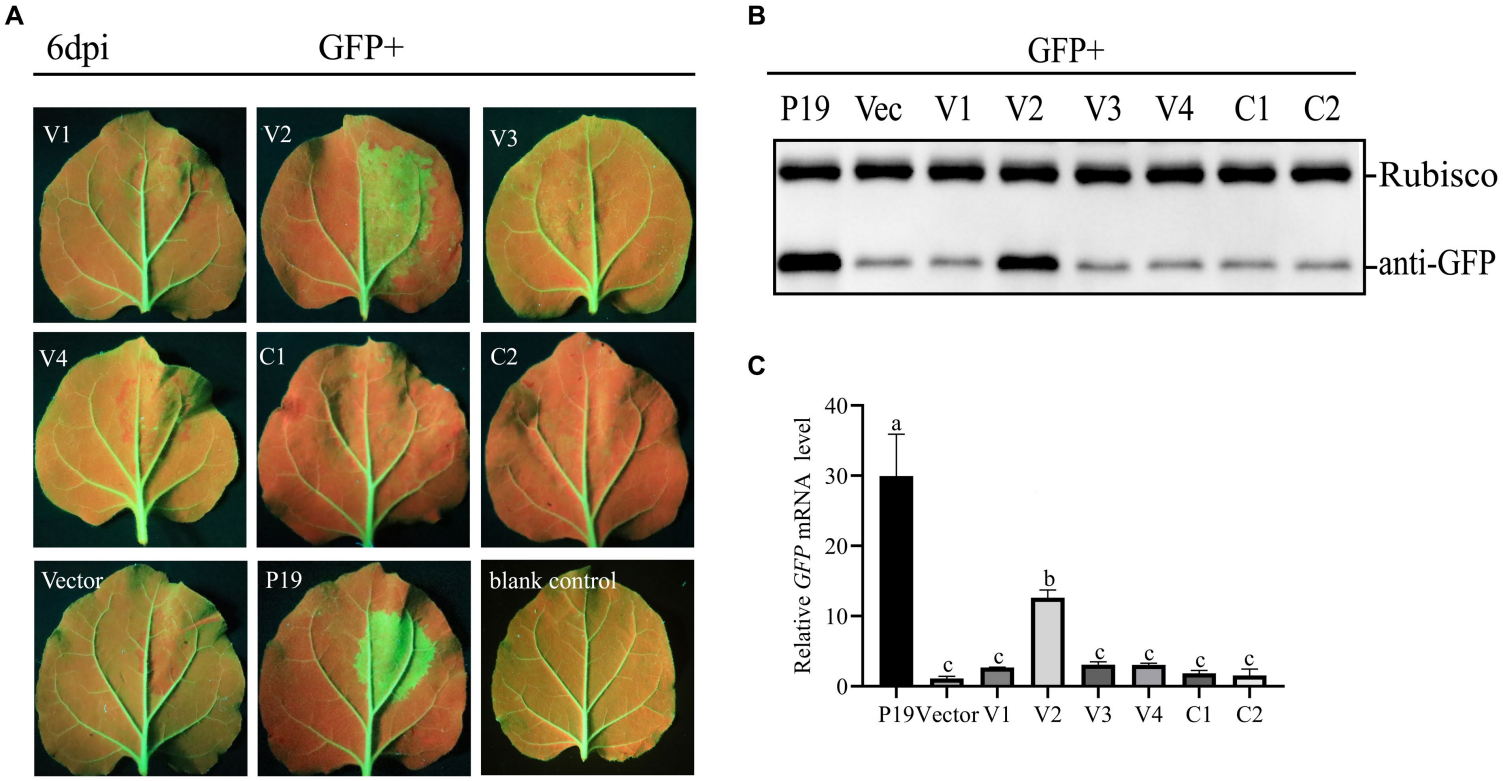
Identification of the RNA silencing suppression activity of proteins encoded by citrus chlorotic dwarf-associated virus (CCDaV). **(A)** *Nicotiana benthamiana* 16c were co-infiltrated with *Agrobacterium tumefaciens* carrying pART27-eGFP expressing GFP and recombinant pCHF3 vectors expressing individual CCDaV proteins. Infiltrated leaves were observed at 6 days post-infiltration (dpi) using UV light. pCHF3-P19 co-infiltrated with pART27-eGFP and pCHF3 co-infiltrated with pART27-eGFP were severed as positive and negative controls, respectively. **(B)** The accumulation of GFP in infiltrated patches was detected by western blotting using anti-GFP antibody. Rubisco served as loading control for the western blot assay. **(C)** The content of GFP mRNA was quantified using real-time PCR and calculated using the 2^−△△Ct^ method with *NbActin* gene as the internal control. The letters represent significant differences among samples (one-way ANOVA, *p* < 0.05). Each experiment was repeated three times.

To test the ability of CCDaV-encoded proteins to inhibit local GFP silencing, six CCDaV-encoded proteins were transiently expressed in the leaves of 16c plants infiltrated with pART27-GFP. GFP expression in 16c leaves was suppressed by the agro-infiltrating pART27-eGFP. At 2 dpi, GFP fluorescence was evidently observed in all leaves of 16c seedlings under UV light. At 4 dpi, GFP fluorescence became weak in leaves co-infiltrated with pART27-eGFP and the empty pCHF3, as well as the recombinant pCHF3 expressing V1, V3, V4, Rep, and RepA (Figure 1A). The GFP fluorescence signal remained strong in leaves co-infiltrated with pART27-eGFP and pCHF3-V2 or pCHF3-P19. However, the fluorescence in leaves infiltrated with pCHF3-V2 + pART27-eGFP (the negative control) was weaker than in leaves infiltrated with pCHF3-P19 + pART27-eGFP (the positive control). Furthermore, at 6 dpi, increased accumulation of GFP mRNA and protein was confirmed in the leaves of 16c plants co-infiltrated with pCHF3-V2 or pCHF3-P19 along with pART27-eGFP, using RT-qPCR and western blot analysis, respectively (Figures 1B,C). These experiments confirmed that V2 suppressed local RNA silencing in 16c cells, but its activity was weaker than that of P19.

### 3.2 Ccdav V2 does not suppress cell-to-cell movement of silencing signal

**Figure 2:**
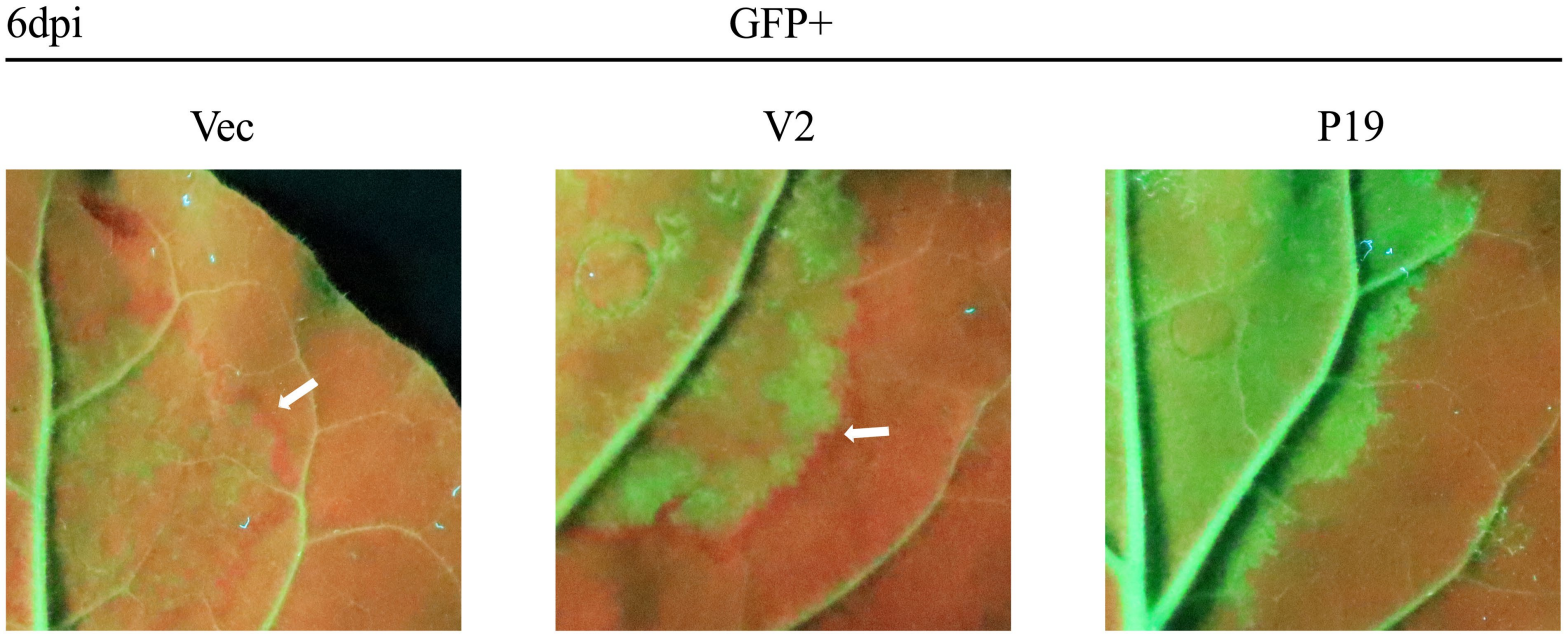
Interference of citrus chlorotic dwarf-associated virus (CCDaV) V2 with intercellular movement of RNA silencing in *Nicotiana benthamiana* 16c. Leaves of 16c were agro-infiltrated with pART27-eGFP plus pCHF3, pCHF3-V2, or pCHF3-P19, respectively. A red ring was observed at 6 days post-infiltration (dpi) under UV light.

A previous study showed that the silenced signal from the infiltrated region transmitted to 10–15 adjacent cells, resulting in reduced GFP expression; a red ring was visible under UV light (Himber et al., 2003). As expected, no red ring was observed around the region co-infiltrated with pCHF3-P19 + pART27-eGFP (Figure 2). However, an obvious red ring developed around the patches co-infiltrated with pCHF3-V2 + pART27-eGFP (positive control) and pCHF3 + pART27-eGFP (negative control). This result demonstrates that V2 could not suppress the intercellular movement of the silencing signals.

### 3.3 V2 inhibits systemic silencing of GFP

**Figure 3:**
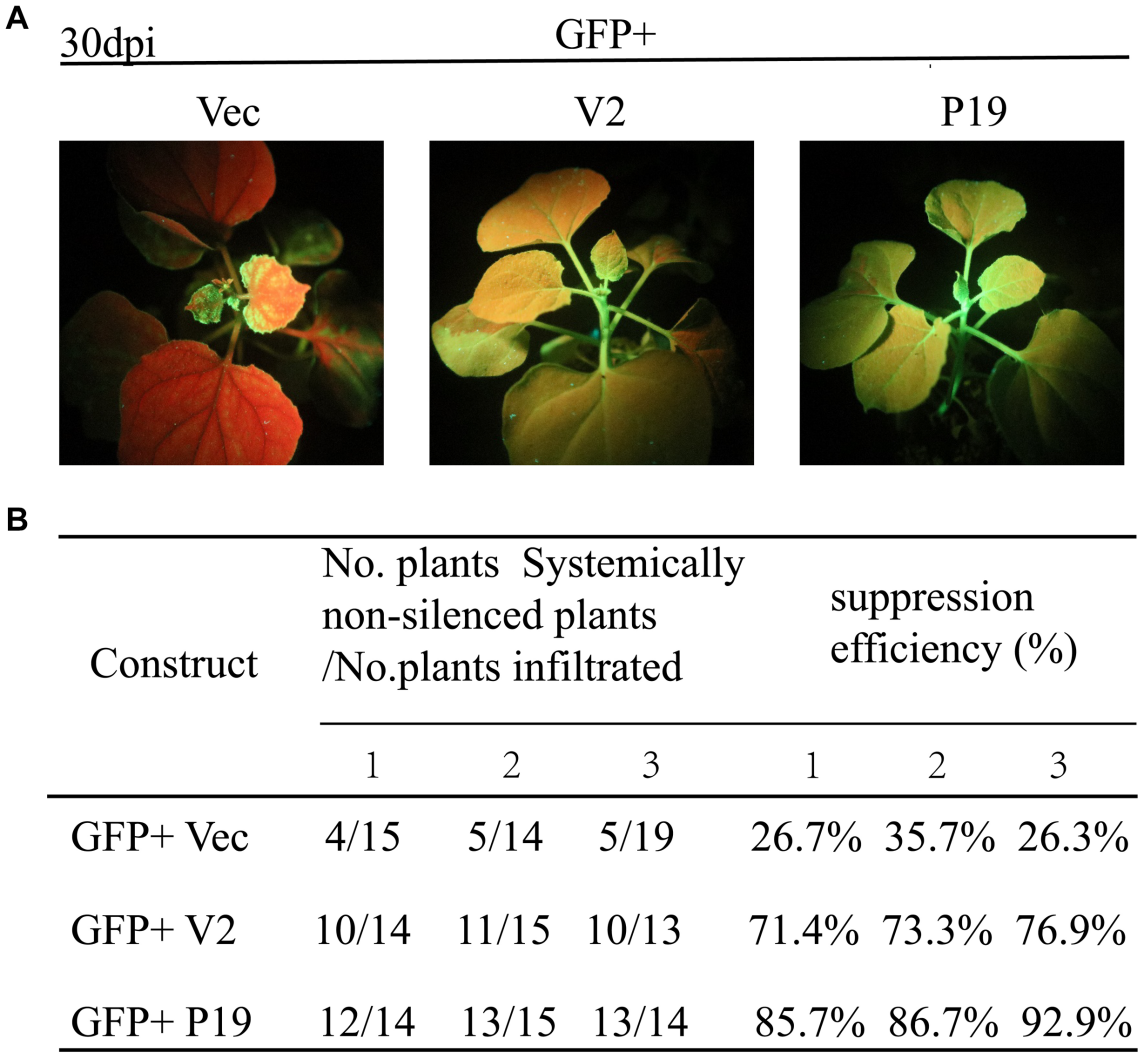
Citrus chlorotic dwarf-associated virus (CCDaV) V2 can suppress systemic silencing. **(A)** *Nicotiana benthamiana* 16c was co-infiltrated with *Agrobacterium tumefaciens* cultures carrying pART27-eGFP plus pCHF3, pCHF3-V2, or pCHF3-P19. The GFP fluorescence was observed at 30 days post-infiltration (dpi) under UV light. 16c seedlings infiltrated with pART27-eGFP and pCHF3-P19 were used as positive control. 16c seedlings infiltrated with pCHF3 and pART27-eGFP were used as negative control. **(B)** Efficiency of silencing induced by V2 in the *N. benthamiana* 16c plant system.

The ability of CCDaV V2 to inhibit systemic silencing induced by GFP in 16c plants was also investigated. At 30 dpi, the new flushes of 16c plants co-infiltrated with pCHF3-V2 and pART27-eGFP showed green fluorescence under UV light, consistent with plants co-infiltrated with pCHF3-P19 and pART27-eGFP (Figure 3A). These results suggested that V2 suppressed the systematic movement of RNA silencing. Furthermore, 73.9% of the 16c plants co-infiltrated with pART27-eGFP and pCHF3-V2 suppressed the movement of *GFP* silencing in systemic leaves, which was lower than that of plants co-infiltrated with pART27-eGFP and pCHF3-P19 (88.4%) (Figure 3B).

### 3.4 V2 cannot block dsRNA-triggered RNA silencing

**Figure 4:**
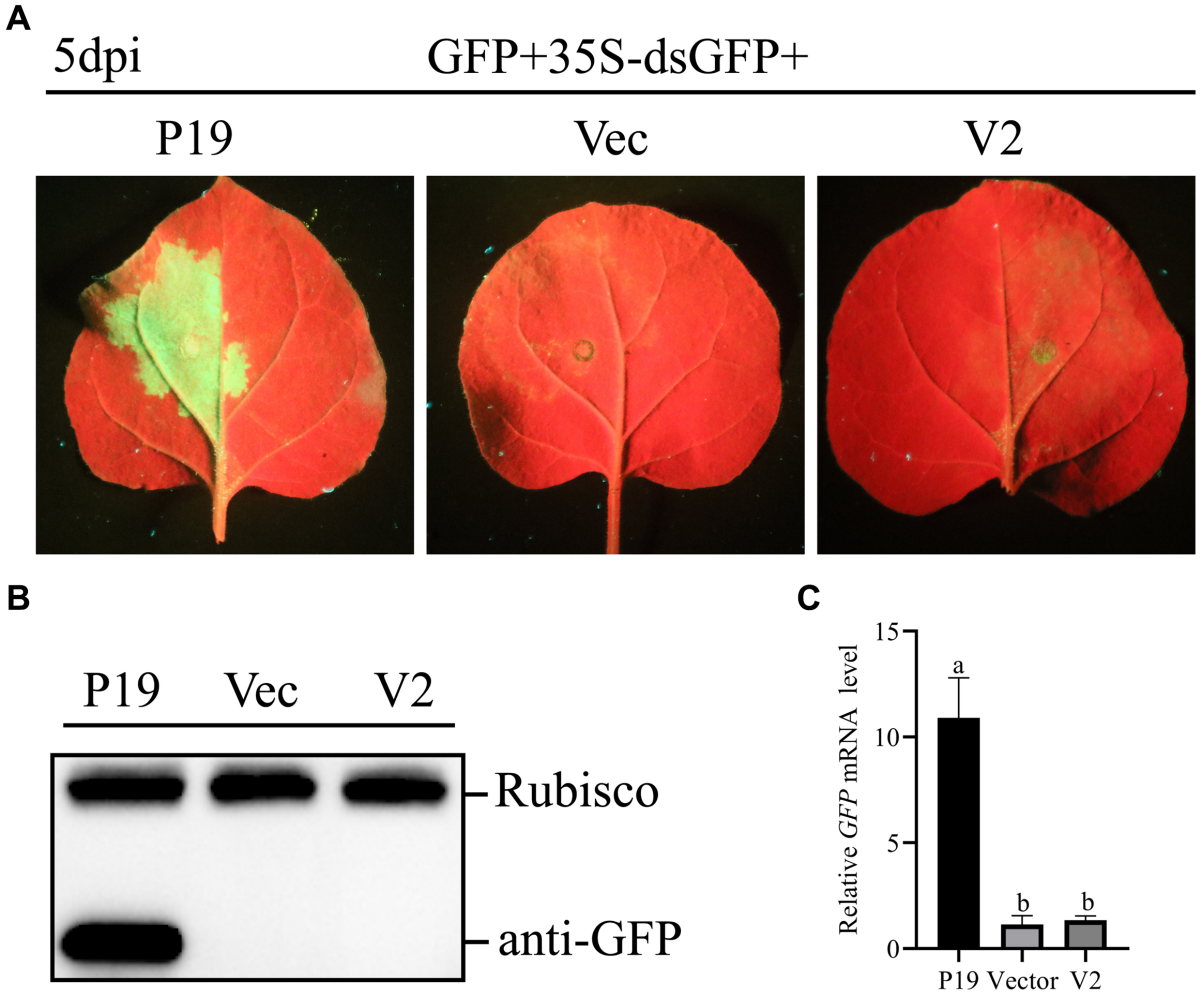
CCDaV V2 could not suppress RNA silencing triggered by dsRNA. **(A)** Representative 16c leaves were co-infiltrated with *Agrobacterium tumefaciens* cultures carrying pART27-eGFP, 35S-dsGFP and pCHF3, or pCHF3-V2 or pCHF3-P19. Photographs were taken at 5 dpi. **(B)** The accumulation of GFP was detected by western blot at 5dpi, and Rubisco served as loading control. **(C)** The content of GFP mRNA was tested by qRT-PCR. *NbActin* was utilized as the internal control. The letters represent significant differences among samples (one-way ANOVA; *p* < 0.05).

To study whether V2 could inhibit dsRNA induced RNA silencing, *A. tumefaciens* GV3101 carrying 35S-GFP + 35S-dsGFP (kindly provided by Professor Jian Ye from the Ningbo University, China), along with empty pCHF3, and constructions carrying V2 and P19 were co-infiltrated into leaves of *N. benthamiana*. As shown in Figure 4A, at 5 dpi, leaves co-infiltrated with pCHF3 + 35S-GFP + 35S-dsGFP or pCHF3-V2 + 35S-GFP + 35S-dsGFP lost green fluorescence, whereas leaves co-infiltrated with pCHF3-P19 + 35S-GFP + 35S-dsGFP showed strong GFP fluorescence. As shown in Figures 4B,C, no GFP protein or *GFP* transcript was detected in leaves co-infiltrated with pCHF3 + 35S-GFP + 35S-dsGFP or pCHF3-V2 + 35S-GFP +  35S-dsGFP. However, GFP protein or *GFP* transcript was detected in leaves co-infiltrated with pCHF3-P19 + 35S-GFP + 35S-dsGFP. These results suggested that V2 could not block dsRNA-induced RNA silencing.

### 3.5 Subcellular localization of V2

**Figure 5:**
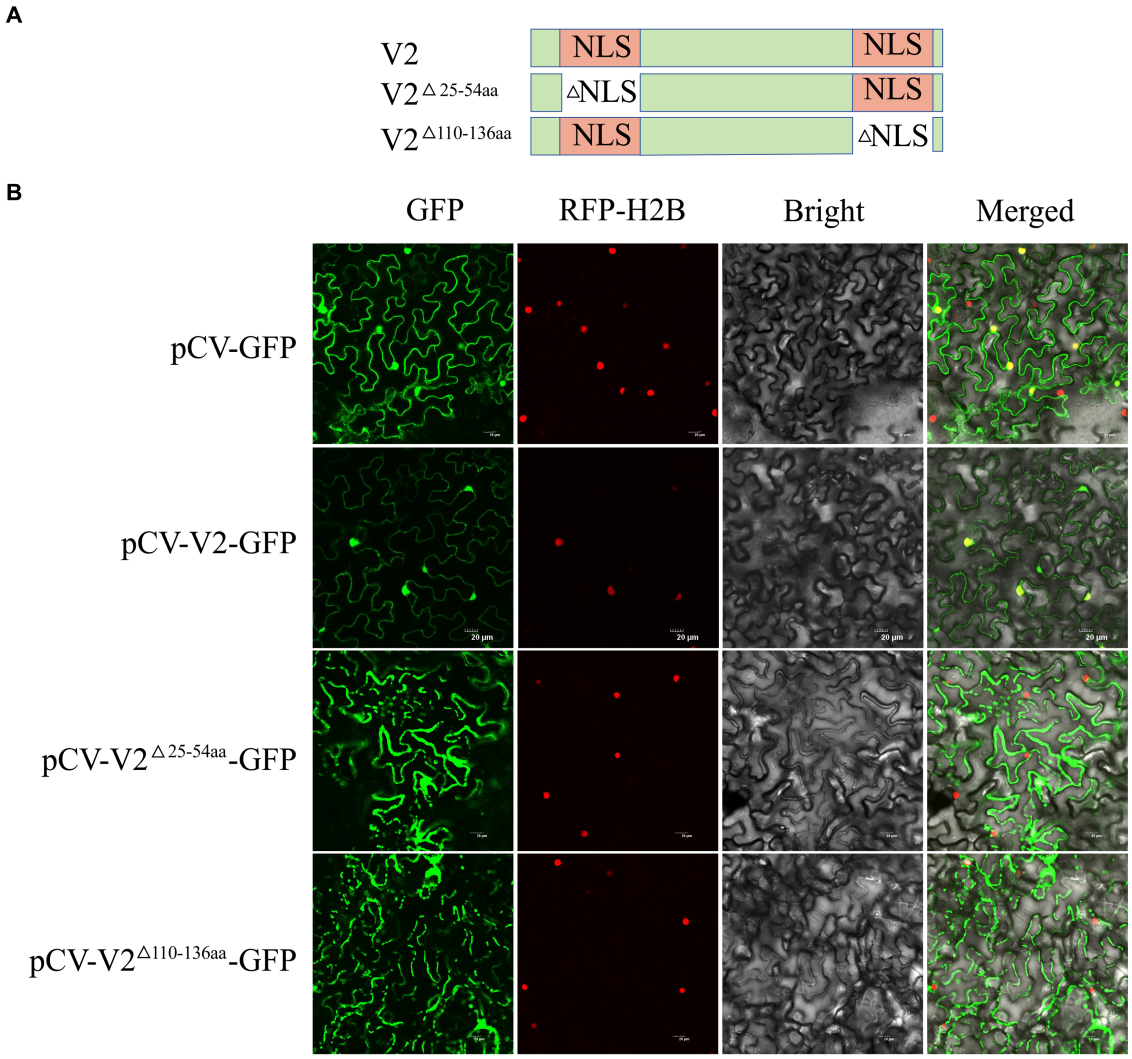
Subcellular localization of CCDaV V2, and its two deletion mutants. **(A)** Schematic representation of CCDaV V2 and two nuclear localization signal (NLS) deletion mutants. **(B)** Red fluorescent protein-histone 2B (RFP-H2B) was co-infiltrated with pCV-GFP, pCV-V2-GFP, pCV-V2^△110-136aa^-GFP, or pCV-V2^△25-54aa^-GFP into *Nicotiana benthamiana* leaves. Scalebar = 20 μm.

Cell-PLoc 2.0 analysis indicated that V2 may be located in the nucleus. To further verify the cellular distribution of V2, pCV-V2-GFP was infiltrated into *N. benthamiana* with mCherry-H2B. At 2 dpi, pCV-V2-GFP was detected in the nuclei of *N. benthamiana.* Analysis using cNLS Mapper software suggested that V2 contains two NLS, comprising 30 aa residues (aa 25–54) in the N-terminal region and 27 aa residues (aa 110–136) in the C-terminal region (Figure 5A). To verify the effect of the putative NLS on the localization ability of V2, two V2 mutants (V2^△25-54aa^ and V2^△110-136aa^) with deleted NLS were constructed and recombined into the pCV-GFP vector. These recombinant plasmids were co-infiltrated into *N. benthamiana* with a nuclei marker, as described above. These results showed that pCV-V2^△25-54aa^-GFP and pCV-V2^△110-136aa^-GFP were not located on the nuclei of *N. benthamiana,* suggesting that aa 25–54 and aa 110–136 are associated with the nuclear localization ability of V2 (Figure 5B).

### 3.6 NLS of V2 involved in suppressing RNA silencing

**Figure 6:**
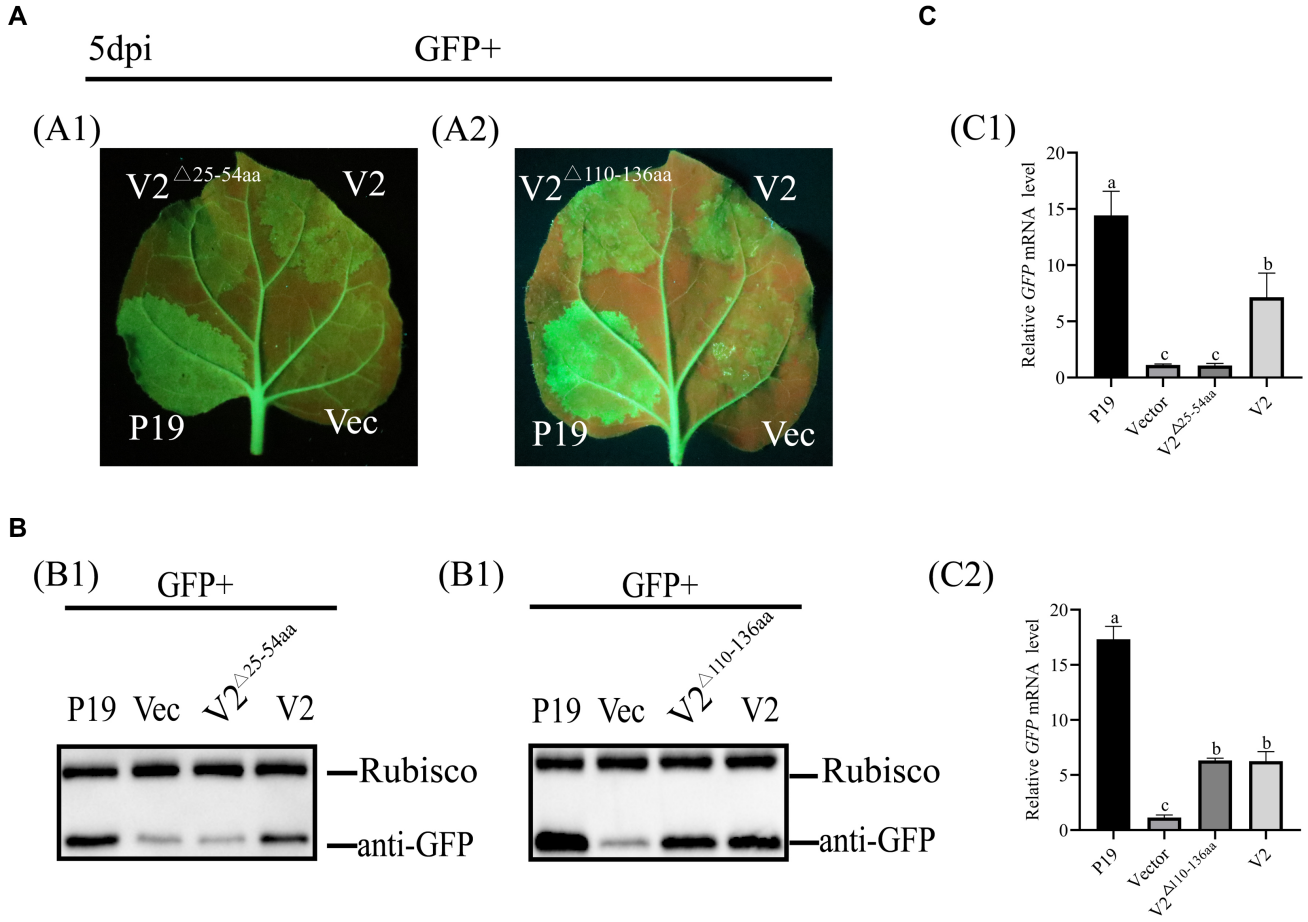
Identification of the NLS of V2 with RNA silencing suppression activity. **(A)** Leaves were co-infiltrated with *Agrobacterium tumefaciens* cultures harboring pART27-eGFP + P19, pART27-eGFP + pCHF3-V2, pART27-eGFP+ pCHF3- V2^△25-54aa^, pART27-eGFP+ pCHF3- V2^△110-136aa^, and pART27-eGFP+ empty vector. The leaves were photographed under UV light at 5 dpi. **(B)** The accumulation of GFP was detected by western blot at 5dpi, Rubisco served as the loading control. **(C)** The content of GFP mRNA was tested by qRT-PCR. *NbActin* was utilized as the internal control. The letters represent significant differences among samples (one-way ANOVA; *p* < 0.05).

To explore the NLS of V2 involved in its ability of RNA silencing, V2^△25-54aa^ and V2^△110-136aa^ were recombined into a pCHF3 vector and used to co-infiltrate with pART27-eGFP into 16c leaves, respectively, as described above. At 5 dpi, no GFP fluorescence was observed in patches co-infiltrated with pCHF3-V2^△25-54aa^ + pART27-eGFP and pCHF3 + pART27-eGFP. Patches co-infiltrated with pCHF3-V2^△110-136aa^ + pART27-eGFP showed GFP fluorescence, but fluorescence was weaker than patches co-infiltrated with pCHF3-P19 + pART27-eGFP (Figure 6). These results suggested that the N-terminal 25–54 aa of V2 involved in suppressing RNA silencing.

### 3.7 Conservation and phylogenetic analyses of V2

**Figure 7:**
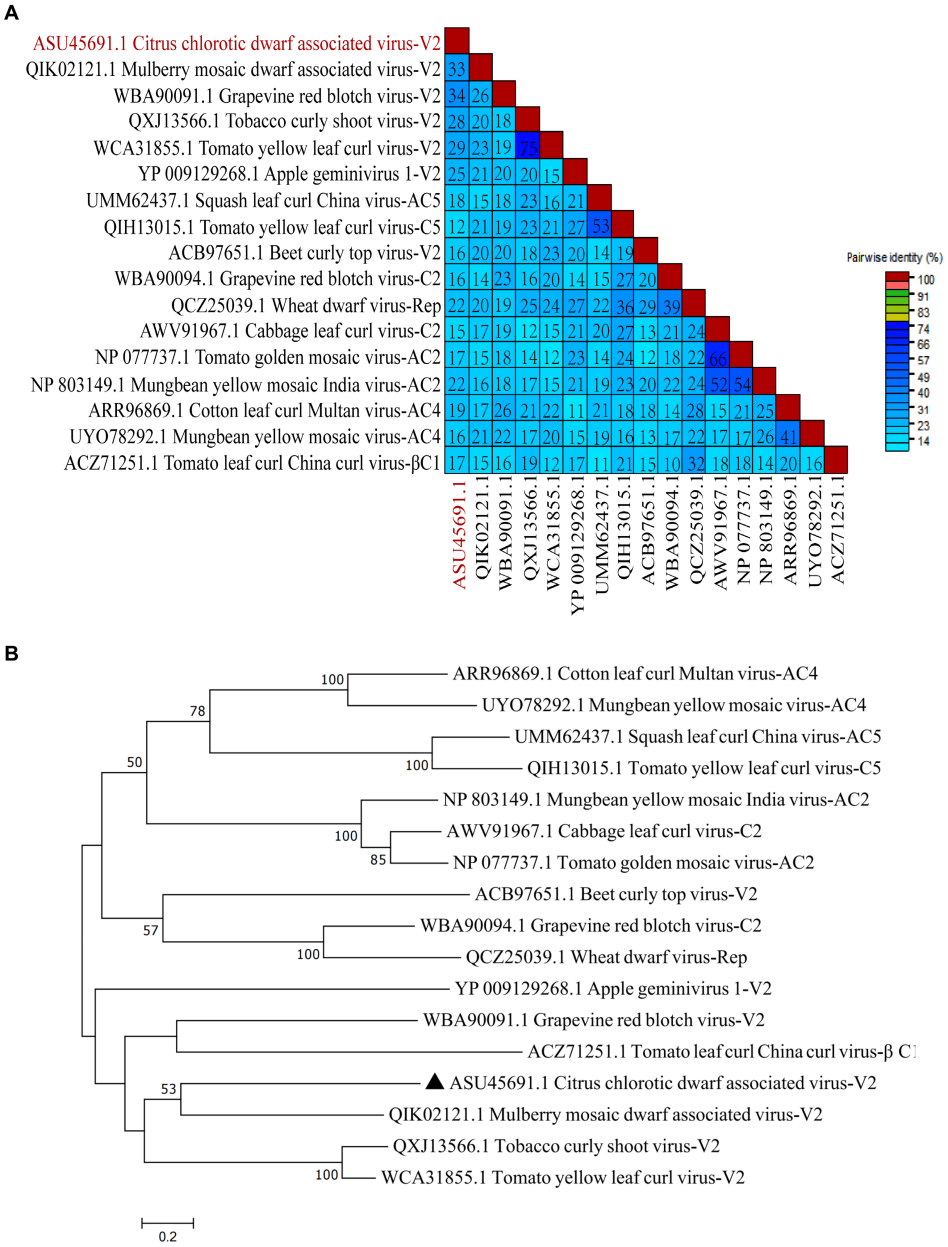
Phylogenetic analysis and pairwise identity of geminivirus V2 protein. **(A)** Pairwise identity matrix of amino acid sequences of RNA silencing suppressors (RSSs) in the Geminiviridae family using SDT v1.2. **(B)** Phylogenetic tree of geminivirus RSSs sequence constructed by the neighbor-joining method with bootstrap 1, 000 repetitions.

To study the conservation of RSSs in *Geminiviridae*, multiple amino acid sequence alignments of 17 RSSs from *Geminiviridae* were performed. The results suggest that CCDaV shares low amino acid similarity with other RSSs from *Geminiviridae* (12.10–33.60%) (Figure 7A). Phylogenetic analysis suggested that CCDaV grouped with mulberry mosaic dwarf-associated virus (MMDaV) (QIK02121.1) to form a distinct clade (Figure 7B).

## 4 Discussion

CCDaV, first recognized in 2012, is an economically important citrus virus, leading to a potential yield loss. However, the mechanism that CCDaV induces disease and interacts with host defense reaction remains unknown. RNA silencing is involved in many processes in the life cycle of a plant. RNA silencing not only plays a significant role in plant growth, development, and response to abiotic stresses, but also is a crucial antiviral defense response in plants (Akbar et al., 2022). To interfere with the different stages of the antiviral RNA-silencing mechanism, many plant viruses have evolved specialized proteins such as RSS to facilitate viral replication, movement, infection, and pathogenicity in plants (Csorba et al., 2015). Furthermore, the same RSS can target multiple stages in this pathway (Orfanidou et al., 2019). In this study, the potential RSS abilities of six proteins encoded by CCDaV were screened by co-agroinfiltration with pART27-eGFP in 16c plants; CCDaV-V2 exhibited the ability to suppress local and systemic RNA silencing. V2 could not suppress intercellular movement of RNA silencing, indicating that the ability of V2 to suppress PTGS is not through direct binding of siRNAs (Wang et al., 2022). Some studies also showed that V2 encoded by MMDaV, along with p25 and p37 encoded by firespike leafroll-associated virus (FLRaV), could suppress local RNA silencing and long-distance movement of the silencing signal in 16c plants, while not affecting short-distance movement of the silencing signal (Yang et al., 2018; Wang et al., 2022). These results are similar to the results of the present study, and this finding enriches our understanding of the functions of proteins encoded by geminiviruses. Furthermore, as CCDaV V2 does not suppress local silencing induced by dsRNA, it may be similar to MMDaV V2 and strawberry vein banding virus (SVBV) P6, targeting the upstream steps of dsRNA generation to prevent the production of siRNA in RNA silencing (Feng et al., 2018; Yang et al., 2018).

Subcellular localization of RSS is associated with its ability to suppress PTGS. Previous studies showed that the NLS of some RSSs, such as cucumber mosaic virus 2b, SVBV P6, and MMDaV V2, is involved in their RNA silencing suppression activities (González et al., 2010; Feng et al., 2018; Yang et al., 2018). In this study, CCDaV V2 distributed in the nucleus of *N. benthamiana* and was predicted to contain two NLSs. The absence of NLS at the N-terminal of V2 resulted in the alteration of its subcellular localization and abolishing its activity of RSS. These results also indicated that V2 may have a nucleocytoplasmic shuttling capability similar to squash leaf curl China virus (SLCCNV) AC5 to regulate the replication of CCDaV in plants (Wu et al., 2022).

Previous studies suggested that RSS of geminiviruses, such as AC5 of SLCCNV (Wu et al., 2022) and mungbean yellow mosaic India virus (MYMIV) (Li et al., 2015), V2 of soybean geminivirus A (SGVA) (Li et al., 2022), apple geminivirus (AGV) (Zhan et al., 2018), and croton yellow vein mosaic virus (CYVMV) (Zhai et al., 2022), also functions as pathogenicity determinants. However, other study showed that although RSS βC1 is involved in the pathogenicity of some begomoviruses, βC1 is not a pathogenicity factor to CYVMV infection (Zhai et al., 2022). Pathogenicity determination assays showed that V2 of CCDaV did not induce apparent symptoms in *N. benthamiana*, suggesting that V2 may have little relevance to viral pathogenicity (Qin et al., 2023). The detailed function of V2 proteins in the pathogenesis of CCDaV requires further study.

In the present study, GFP fluorescence in 16c leaves co-infiltrated with pCHF3-V2 + pART27- eGFP was stronger than in leaves co-infiltrated with pCHF3-P19 and pART27-eGFP. This result indicated that the ability of V2 to suppress silencing was weaker than that of P19. However, a previous study suggested that V2 is the strongest PTGS suppressor encoded by begomoviruses and that its silencing ability is similar to P19 (Luna et al., 2017). This discrepancy may be related to the low sequence similarity between CCDaV V2 and RSSs encoded by other geminiviruses.

## 5 Conclusion

V2 of CCDaV functions as an RSS to interfere with the local RNA silencing and systemic movement of the silencing signal. To the best of our knowledge, this study is the first to identify the RSS of *Citlodavirus.* Further studies should focus on host factors that interact with V2, which may provide new strategies for controlling CCDaV.

## Data availability statement

The original contributions presented in the study are included in the article/Supplementary material. Further inquiries can be directed to the corresponding authors.

## Author contributions

XY: Writing – original draft, Data curation, Formal analysis, Investigation, Methodology. DD: Investigation, Writing – original draft. YC: Formal analysis, Writing – original draft. CL: Investigation, Writing – original draft. ZL: Designed the experiments, Analyzed the data. BL: Methodology, Writing – review & editing. YZ: Methodology, Writing – review & editing.

## Footnotes

^1^http://www.csbio.sjtu.edu.cn/bioinf/Cell-PLoc-2/

^2^http://nls-mapper.iab.keio.ac.jp/cgi-bin/NLS_Mapper_form.cgi
