# Supplementary material for: Identification of RNA silencing suppressor encoded by citrus chlorotic dwarf-associated virus
Source: Front Microbiol. 2024 Jan 25;15:1328289. doi: 10.3389/fmicb.2024.1328289 (PMC10850569; doi:10.3389/fmicb.2024.1328289)
Supplement: Supplementary file 1 [file Table_1.DOCX]

Supplementary Material

# Supplementary Tables

| Primers | **Sequence (5' –3' )** |
| --- | --- |
| pCHF3-V1-F | ACGAGCATCTAGGTCGACAATGGTGAGTACCAGGAGTGGA |
| pCHF3-V1-R | TACCGCATCCCGGGTACCATTAATTTGATGTAGAATCATAAAAATACAATCGAGCCTG |
| pCHF3-V2-F | ACGAGCATCTAGGTCGACAATGTGTCATTATGCATTAAGTGTTCAAGATTTG |
| pCHF3-V2-R | TACCGCATCCCGGGTACCATTACACCCCGGAGGAACAC |
| pCHF3-V3-F | ACGAGCATCTAGGTCGACAATGAAACGTGTTGGGCACG |
| pCHF3-V3-R | TACCGCATCCCGGGTACCATTACCCAGGGCTCCTCCTA |
| pCHF3-V4-F | ACGAGCATCTAGGTCGACAATGGACGGTCAAGATTTGGTGTTACA |
| pCHF3-V4-R | TACCGCATCCCGGGTACCATTAAGCCAAGCTTTTTTTCTTATAATCATTAGCATTAC |
| pCHF3-C1-F | ACGAGCATCTAGGTCGACAATGGCTTCCACTTCCTCTAGCT |
| pCHF3-C1-R | TACCGCATCCCGGGTACCACTAAACTTCTGGCCCAGGC |
| pCHF3-C2-F | ACGAGCATCTAGGTCGACAATGGCTTCCACTTCCTCTAGCT |
| pCHF3-C2-R | TACCGCATCCCGGGTACCACTAGTAGAATACATCAGTGGGGTCCATATAATGCATAA |
| pCV-V2-F | GAGAACACGGGGGACTCTAGAATGTGTCATTATGCATTAAGTGTTCAA |
| pCV-V2-R | ATCCTTGTAATCCATTCTAGACACCCCGGAGGAACACCTT |
| pCV-V2^△25-54aa^-2-F | GTTGAGTGTGCTTATACGGTTAATACGTAGATACTGCG |
| pCV-V2^△25-54aa^-1-R | ACCGTATAAGCACACTCAACATGCTCATTAAACCG |
| pCV-V2^△V110-136aa^-R | atccttgtaatccattctagaCACCCCGGAGGCCACCTG |
| q-eGFP-F | CTTCTTCAAGTCCGCCATGCC |
| q-eGFP-R | GTTGTGGCGGATCTTGAAGTTC |
| q-NbActin-F | TGGTCGTACCACCGGTATTGTGTT |
| q-NbActin-R | TCACTTGCCCATCAGGAAGCTCAT |
| pCHF3-V2^△25-54aa^-F | ACGAGCATCTAGGTCGACAATGTGTCATTATGCATTAAGTGTTCAAGATTTG |
| pCHF3-V2^△25-54aa^-R | TACCGCATCCCGGGTACCATTACACCCCGGAGGAACAC |
| pCHF3-V2^△V110-136aa^-F | ACGAGCATCTAGGTCGACAATGTGTCATTATGCATTAAGTGTTCAAGATTTG |
| pCHF3-V2^△V110-136aa^-R | TACCGCATCCCGGGTACCATTACACCCCGGAGGCCACCTG |

Table S1 Primers used in this study

**
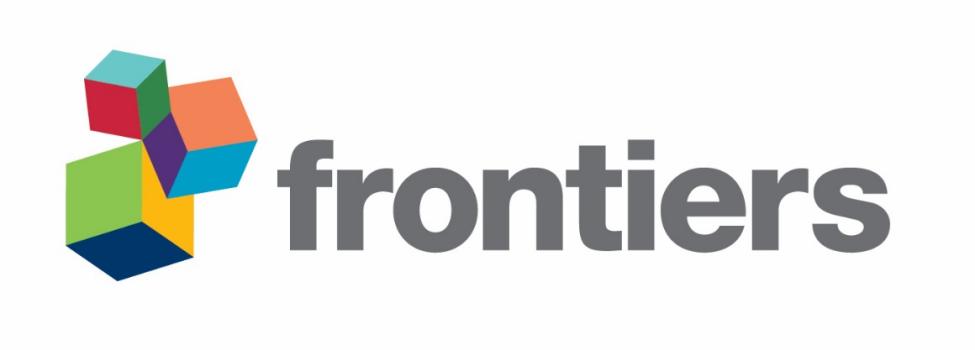
**
